# Evaluation of learning outcomes of humanities curricula in medical students. A meta-review of narrative and systematic reviews

**DOI:** 10.3389/fmed.2023.1145889

**Published:** 2023-04-17

**Authors:** Valle Coronado-Vázquez, Cristina Antón-Rodríguez, Juan Gómez-Salgado, María del Valle Ramírez-Durán, Santiago Álvarez-Montero

**Affiliations:** ^1^B21-20R Group, Instituto Aragonés de Investigaciones Sanitarias, University of Zaragoza, Zaragoza, Spain; ^2^Las Cortes Health Centre, Madrid Health Service, Madrid, Spain; ^3^Faculty of Medicine, Universidad Francisco de Vitoria, Madrid, Spain; ^4^Department of Sociology, Social Work and Public Health, Faculty of Labour Sciences, University of Huelva, Huelva, Spain; ^5^Safety and Health Postgraduate Program, Universidad Espíritu Santo, Guayaquil, Ecuador; ^6^Department of Nursing, University Centre of Plasencia, University of Extremadura, Plasencia, Spain

**Keywords:** medical education, humanities, learning outcomes, undergraduate, university teaching

## Abstract

**Objectives:**

To assess the expected learning outcomes of medical humanities subjects in medical studies curricula. To connect those expected learning outcomes with the types of knowledge to be acquired in medical education.

**Methods:**

Meta-review of systematic and narrative reviews. Cochrane Library, MEDLINE (Pubmed), Embase, CINAHL, and ERIC were searched. In addition, references from all the included studies were revised, and the ISI Web of Science and DARE were searched.

**Results:**

A total of 364 articles were identified, of which six were finally included in the review. Learning outcomes describe the acquisition of knowledge and skills to improve the relationship with patients, as well as the incorporation of tools to reduce burnout and promote professionalism. Programs that focus on teaching humanities promote diagnostic observation skills, the ability to cope with uncertainty in clinical practice, and the development of empathetic behaviors.

**Conclusion:**

The results of this review show heterogeneity in the teaching of medical humanities, both in terms of content and at the formal level. Humanities learning outcomes are part of the necessary knowledge for good clinical practice. Consequently, the epistemological approach provides a valid argument for including the humanities in medical curricula.

## Introduction

Since medicine is a human-applied science, physicians should acquire specific scientific knowledge, technical skills, and attitudes to practice medicine in compliance with ethics and professionalism. All these aspects should be taken into consideration when defining the expected learning outcomes and the ways of evaluating them within medical education.

Humanities have been increasing their relevance in medical studies over the years, providing students with a more critical and reflective thought process which can help them to offer better and more context-adapted care to their patients (1). Furthermore, humanities have also meant a solution to the shortcomings of a positivist orientation of medicine.

Since humanities contribute to the building of medical knowledge, it is essential to reflect on them and to determine how to embed them in the curricula. However, it is not an easy task to perform as humanities encompass various disciplines, including classical humanities, i.e., philosophy and history, and social sciences, i.e., anthropology and arts (2). These are considered to enhance observational skills, which are essential for diagnosis and clinical decision-making processes (3).

Regarding its proven contribution to medical studies, several approaches have been developed to include humanities in medical curricula. From an instrumental approach, humanities are required to develop clinical observational skills, empathy, and communication skills. On the other hand, the non-instrumental approach bases its argument on stating that humanities have a value of their own and emphasize their capacity for personal development and self-understanding. These abilities contribute to the characteristics of a good physician who, in addition to having scientific skills and knowledge, need to be able to understand people (4). In consequence, both humanities and sciences would be integrated into clinical judgement and converge in the way they interpret objective data within the patient’s psycho-social context.

From an epistemological approach, humanities are part of a body of knowledge which is paramount to provide professional clinical practice. This knowledge is rational, interpretative, logical, intuitive, partly predictable yet fundamentally uncertain (5). Kumagai (6) establishes three types of medical education, namely technical, practical, and critical knowledge. This division has its origin in the interests that, according to Habermas (7), guide the knowledge of human beings depending on whether the interest is in manipulating reality (empirical sciences), accessing the meaning of cultural realities (social sciences), or criticizing and modifying reality. In the medical field, technical knowledge would be represented in biomedical areas, while practical knowledge would include diagnostic and treatment standards established by expert consensus; critical knowledge would, in turn, seek to understand people’s problems in a reflective way in order to integrate the acquired knowledge into clinical practice, so humanities would be included in this type of critical knowledge (6).

Even though humanities have been progressively introduced in Health Sciences curricula, there is a lack of homogeneity as regards content and the description and assessment of the expected learning outcomes; instead, there exists a promotion of the justification of including humanities in Health Sciences rather than evidence of their impact on students’ learning outcomes (8). As a result, Health Sciences studies’ curricula present heterogeneity, which obstructs their unified evaluation. For instance, Howick et al. (9) assessed medical schools curricula in Canada, the United Kingdom, and the United States revealing that the most frequently taught humanities subjects were history and literature or narrative medicine, sociology and social medicine, arts, medical humanities, philosophy, and theology. Studies such as this one reveal that the inclusion of these subjects improves clinical judgement (5) and professionalism (10), among other medical competencies. Furthermore, Orefice et al. (11) analyzed several medical colleges’ curricula from Spain and Italy and found that the most frequently taught subjects related to humanities were medicine history and bioethics. However, the assigned European credit transfer and accumulation system (ECTS) to those subjects were scarce compared to what is being allocated to more medical-oriented subjects (11). This fact hampers the aim of providing a holistic view of illness that merges scientific knowledge with a conscience of what can and should be done.

In United States universities, it is also common to find humanity programs based on narrative medicine, such as at Columbia University, where literary theory, philosophy, ethics, and the arts are included (12).

A student-centered approach to education can be enhanced by learning outcomes. They provide clarity and transparency, facilitating the integration of subjects and allowing for international comparisons (13). They also help to establish the definition of objectives, methodologies, and assessment methods.

Various organizations and authors have offered different definitions of learning outcomes. The definition from The Framework for Qualifications of the European Higher Education Area was followed in this case, where learning outcomes are defined as ‘statements of what a learner is expected to know, understand, and/or be able to do at the end of a period of learning’ (14).

Specifying medical humanities’ learning outcomes would provide purpose, consistency, and justification. Hence the interest in identifying explicit learning outcomes from these subjects. Two questions were posed:

1.What are the expected learning outcomes of medical humanities subjects? How are they assessed?

2.How do humanities learning outcomes relate to the types of knowledge to be acquired in medical education?

## Aims

1. To assess the expected learning outcomes of medical humanities subjects in medical studies curricula.
2. To connect those expected learning outcomes of medical humanities subjects with the types of knowledge to be acquired in medical education.

## Methods

### Study design

Meta-review of systematic and narrative reviews was carried out. Meta-reviews highlight the most important points and integrate information from systematic reviews and other types of studies. Systematic or narrative reviews may include randomized clinical trials, cohort or case–control studies, cross-sectional, quasi-experimental, case reports, or qualitative studies.

Systematic review methods were followed to locate, analyze, and synthesize the available information. Furthermore, the PRISMA (*Preferred Reporting Items for Systematic Reviews and Meta-Analyses)* declaration was followed to plan and divulge the findings (15). Afterwards, findings were discussed according to types of knowledge to be acquired in medical education.

### PIO format

- Participants: Medicine and Health Sciences students.
- Intervention: Humanities subjects in undergraduate degrees.
- Outcomes: Expected learning outcomes, defined as the skills and knowledge that students are expected to achieve at the end of a subject learning process.

### Search strategy

The following databases were searched until 23 July, 2022: Cochrane Library, MEDLINE (Pubmed), Embase, CINAHL (Cumulative Index to Nursing and Allied Health Literature), and ERIC. Also, references from all the included studies were revised, and the secondary search was broadened by searching ISI Web of Science and Database of Abstracts of Reviews of Effects (DARE).

- Databases search strategies:
- EMBASE: “health humanities” AND (“curriculum”/exp. OR “curriculum”) AND (“evaluation”/exp. OR “evaluation”).
- Pubmed: (“health”[MeSH Terms] OR “health”[All Fields]) AND (“humanities”[MeSH Terms] OR ‘humanities”[All Fields]) AND (“education”[Subheading] OR “education”[All Fields] OR “curriculum”[All Fields] OR “curriculum’[MeSH Terms]) AND “evaluation”[All Fields] AND (“education”[Subheading] OR “education”[All Fields] OR “educational status”[MeSH Terms] OR (“educational”[All Fields] AND “status”[All Fields]) OR “educational status”[All Fields] OR “education”[All Fields] OR “education”[MeSH Terms]) AND (“medicine”[MeSH Terms] OR “medicine”[All Fields]).
- Cochrane, CINAHL, ERIC, DARE, and WOS: MESH “Health humanities”, “Curriculum”, “Evaluation”.

Results were filtered by English and Spanish languages.

### Inclusion and exclusion criteria

Following the standards for evidence synthesis, the inclusion criteria were determined according to the PICO terms (population, intervention, comparison, outcome).

- Inclusion criteria:

Type of study: Narrative and systematic reviews.

Population: Medical and other Health Sciences students.

Intervention: Humanities studies in medical and other Health Sciences curricula.

Comparison: The studies included in the reviews may or may not have a control group.

Outcome: Interventions were measured in terms of expected learning outcomes.

- Exclusion criteria:

Systematic or narrative reviews that did not address the expected learning outcomes of medical humanities subjects were excluded.

### Data extraction

**Figure 1:**
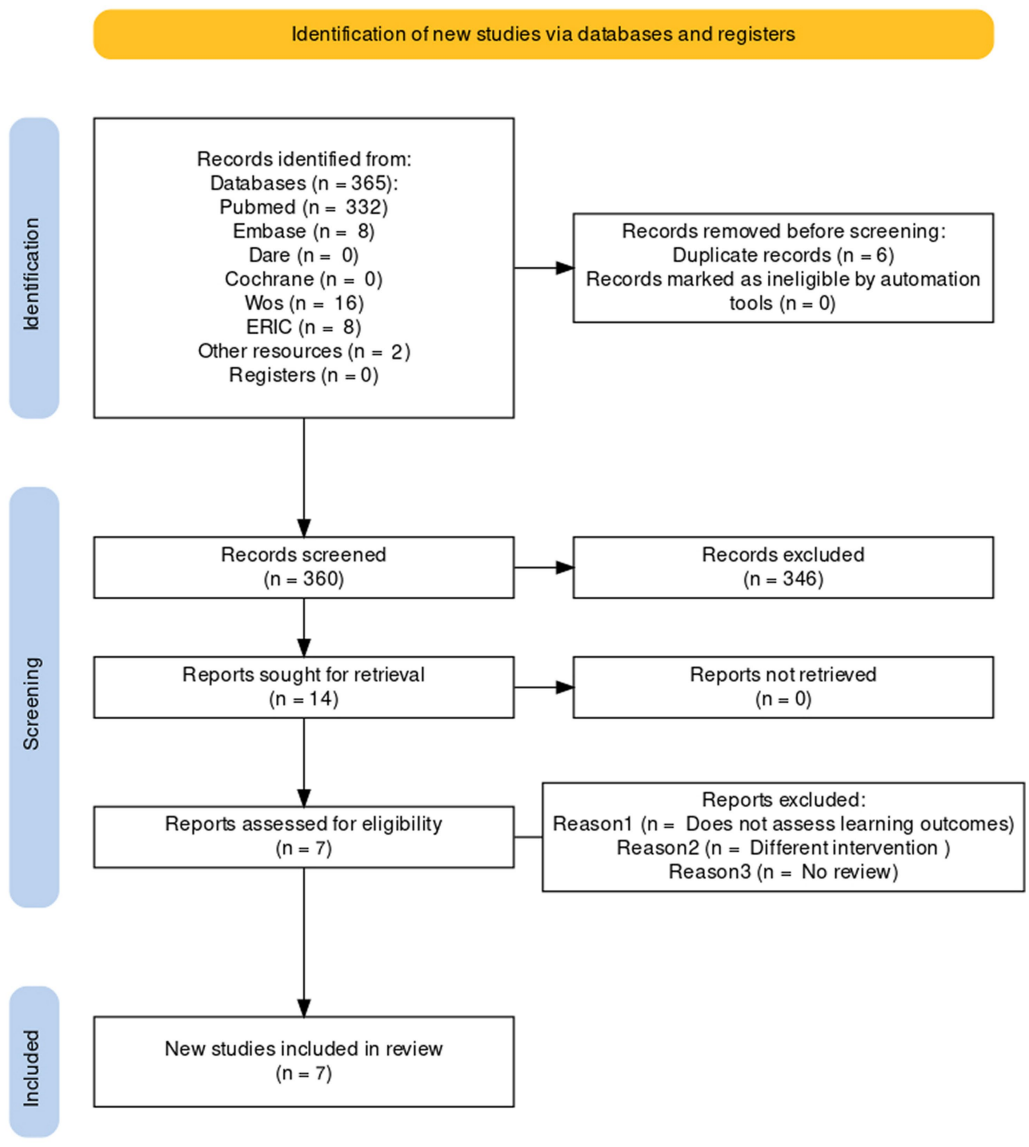
PRISMA flow diagram (Spain, 2022).

Study selection and data extraction were performed by two reviewers. Discrepancies were resolved by discussion and agreement. In the first phase, the titles of the articles found in the databases were examined, and duplicates were removed. Next, the abstracts were read; those that met the inclusion criteria were selected and the full texts of all of them were obtained. After this reading process, the final studies were selected and included in the meta-review (Figure 1). Data on the year of publication, type of study, population, medical humanities programs, learning outcomes, evaluation, and evidence of results were extracted.

### Critical appraisal

GRADE-CERQual was used for narrative reviews (16), and AMSTAR 2 for systematic reviews (17).

GRADE-CERQual (Confidence in the Evidence from Reviews of Qualitative research) assesses the confidence of the evidence through four components:

- Methodological limitations of the included studies: To what extent are there concerns about the design or conduct of the primary studies?
- Coherence of review findings: How clear and cogent is the fit between the data from the primary studies and a review finding?
- Adequacy of data: Are the data adequate in quantity and richness?
- Relevance: To what extent are data from the primary studies that support a review finding applicable to the context (perspective or population, phenomenon of interest, setting) specified in the review question?

Confidence levels were established according to whether the results of the review represented the objective of study: High (very likely to represent it); Moderate (likely); Low (possible); Very low (it is not clear that the results represent the phenomenon under study).

AMSTAR 2 consists of 16 domains. Domains 2, 4, 7, 9, 11, 13, and 15 are considered critical for establishing the assessment of the confidence of the review. This confidence can be high (no critical weakness and up to one non-critical weakness); moderate (more than one non-critical weakness); low (one critical flaw with or without non critical weaknesses); and very low (more than one critical flaw with or without non-critical weaknesses).

### Data synthesis

A description of the data extracted from the articles was made, collecting the following data:

- Article citation: Authors, year of publication.
- Type of review.
- Country.
- Population: Medical and other Health Sciences students.
- Interventions: Type of educational intervention.
- Learning outcomes defined as the acquisition of skills, abilities, and attitudes.
- Evaluation of the learning outcomes. Learning outcomes were discussed according to the types of knowledge to be acquired in medical education established by Kumagai (6).

## Results

A total of 360 research papers were identified (Figure 1), of which seven (18–24) were included in the review (Table 1). Medical students were the studied population in three articles (18, 22, 23), whereas other Health Sciences students, including nurses and odontology students, were under study in the rest (19–24). Of the included studies, two were systematic reviews (19, 24) and the rest were narrative reviews. There was variability among the taught medical humanities, where philosophy, epistemology, anthropology, drama, cinema, history (18, 20, 24), narrative medicine (19, 22), or general arts such as poetry, theater, or painting (21, 23) were studied. Literature or narrative medicine were most stated. A lack of homogeneity in the duration of sessions was found, being the range from a few hours to a whole academic year (19, 21).

**Table 1:** Studies included in the review (Spain, 2022).

Studies	Population	Country	Type of study	Intervention: Medical humanities curriculum	Learning outcomes	Learning assessment	Evidence of the results
Ousager et al. (18) 2010 Narrative review	Medical students	Canada, United States.	Cross-sectional studies, reports, opinions, and assessments.	Philosophy and epistemology. Gender studies and anthropology. Drama, cinema, history, and creative writing.	– Empathy. – Ethical performance. – Better understanding of patients’ problems. – More self-confidence in problem management.	– Course evaluated by students. – Assessment of attitudes toward empathy, spirituality, and tolerance.	Lack of empirical evidence of the long-term impact of humanities in medical education.
Remein (19) 2020 Systematic review	Medical, odontology and nursing students. Medical residents. Physicians and nurses.	United States, Canada, Europe, South/West Asia, South America.	Cross-sectional studies, case–control, pre-post, and randomized studies. Qualitative, quantitative, and mixed evaluation.	55 medical writing programs: – Textual analysis/close reading of published literature. – Creative/reflective writing. – Patient history writing.	– Cultivation of reflection. – Empathy. – Communication, active listening, and narrative competence. – Burnout detection and/or reduction. – Cultural competence. – Narrative skills for pedagogy. – Promoting clinical competence. – Increased sense of professionalism and vocation. – Successful medical teamwork.	– Participant satisfaction. – Pre-post assessment in competencies such as relationship building, empathy, confidence/personal achievement, pedagogical, and clinical skills. – Assessment of narrative competence and communication, ability to detect and mitigate burnout, to encourage reflection on professional identity formation, and promote teamwork.	Evaluation methods and program outcomes were underdeveloped.
Carr et al. (20) 2021 Scoping review	Medical, nursing students, and other health sciences studies.	United States, Canada, United Kingdom, Australia, India, Sweden, Ireland, Spain, New Zealand.	Qualitative and mixed-methods studies.	Arts, literature, practical and reflective narrative, cinema, ethics, and philosophy.	– Patient-centered care skills. – Knowledge to promote humanism. – Personal growth. – Promoting professional well-being. – Capacity for self-reflection or introspection. – Communication and listening.	– Evaluation of student response and satisfaction. – Impact on behavioral change, knowledge, and skills. – Evaluation through reflective and narrative writing.	Lack of consistent description of learning outcomes, making it difficult to compare humanities curricula.
Haidet et al. (21) 2016 Narrative review	Medical, nursing students and other health sciences studies.	United States, Finland, Canada, Netherlands, Australia, United Kingdom, Nepal, Brazil, Sweden.	Empirical and conceptual studies. However, the type of study was not detailed.	Arts, including drama, poetry, dance, music, literature, and visual arts.	– Development of self-awareness. – Ability to cope with uncertainty. – Development of sensitivity and empathy. – Observational skills, communication, critical and creative thinking, and ethical reasoning.	Not declared.	No data were collected regarding the evidence of the results.
Argawal et al. (22) 2015 Narrative review	Medical students	United States, New Mexico, Korea.	Cross-sectional studies.	Optative preclinic courses regarding end-of-life care, creative writing, and literature.	– Empathy improvement. – Better coping strategies for working with end-of-life patients.	– Self-evaluation. – Quantitative measurement of empathy and attitudes toward the humanities.	No data were collected regarding the evidence of the results.
Perry et al. (23) 2014 Narrative review	Medical students	United States, United Kingdom	Not specified. Only one was a randomized clinical trial.	Scenic, mixed, and visual arts. Literature.	– Effects on students’ attitudes. – Effects on behavior were not assessed.	– Nominal groups. – Written assessment. – Qualitative measures of how students understand patients.	Weak evidence due to methodological deficits (methods not well described, evaluations based on students’ opinions).
Hoang et al. (24) 2022 Systematic review	Medical and nursing students. Non-medical/nursing related students	Taiwan	Qualitative, mixed-methods studies and quasi-longitudinal, baseline survey.	Exposure to visual art, elderly community care practice, Field work after informal and formal humanities training, narrative/ Storytelling, course, Reflective writing practice and receiving feedback from mentor, Field practice program, Silent mentor” (death human body) initiation ceremony, Course with problem-based learning, lectures and feedback,	– Participants ‘medical humanities skills development/professional development – Appreciation of the medical humanities – Empathy, professional behavior, intentions for learning or cognitive skills, and mental or psychological health of student – Medical humanities construct	– For the evaluation of participants’ medical humanities skills development/professional development: Self-assessments, faculty observation, scheduled and unscheduled tests, written assignments (self-reflection and feedback), reflective/ narrative writing and quantitative questionnaires. – To assess the appreciation of the medical humanities: participants’ narratives – To assess empathy, professional behavior, intentions for learning or cognitive skills, and mental or psychological health of student: faculty observations of student’s discussions – To assess medical humanities construct: scheduled and unscheduled tests.	Outcomes were assessed using a variety of ways No study reported observable changes in the students’ themselves in terms of application to daily life after their newly acquired knowledge/ attitude, and at organizational or patient levels

Overall, the expected learning outcomes were focused on the acquisition of knowledge and skills toward developing a better professional-patient relationship from different approaches. These outcomes were acquisition of knowledge and skills to better understand and communicate with patients, such as empathy and ethical reasoning, and development and practice of patient-centered care and clinical competence. Another approach regarding outcomes focused on the promotion of professionals’ well-being, including skills for reducing burnout, confronting uncertainty, and promoting professionalism and self-reflection. Regarding clinical competence and teamwork, only Remein et al. (19) reported these expected learning outcomes.

Haidet et al. (21) and Perry et al. (23) applied art-centered programs to enhance creative thinking and promote diagnostic observation and pattern recognition skills, both necessary for clinical diagnosis. Additionally, these skills are also related to the capacity of being able to confront clinical practice ambiguity and develop empathetic behaviors when experiencing the work created by others. In consequence, observation, communication, critical thought, and ethical reasoning are improved (21).

Long-term impacts on clinical practice have been documented in few papers. An explanation for this problem might be the methodological difficulties encountered when designing studies aiming to explain learning outcomes (20).

Regarding the objectives of the interventions, overall, they addressed the domains of knowledge, skills, and attitudes/behavior. Most interventions were intended to develop skills to train students in patient-centered care (20). Promoting reflection, empathy, and communication has been described as a goal in the narrative review by Remein et al. and the systematic review by Hoang et al. (19, 24). Reflective writing in this case was aimed at developing and practicing the skills of reflection so they could be applied to future health care practice (20). Curricula goals were focused on developing students’ capacity for perspective, reflexivity, self-reflection, and person-centered approaches to communication (20).

The evaluation of the assessed humanities programs was mainly carried out through surveys, focus groups, interviews, and reflective writing. It included aspects such as student satisfaction and impact on knowledge, skills, and behaviors (18, 20, 24). It also focused on competencies such as empathy (18, 20, 24), narrative competence, communication, personal development, and clinical skills (19). However, one study did not include an evaluation of the programs (21).

The assessed evaluations have shown evidence of improvement in competencies such as empathy, reflection, resilience, personal development, and clinical skills (19, 24). However, in general terms, the evidence of outcomes in the included articles in all reviews was weak and presented deficits in the assessment methodology and lack of consistent description of learning outcomes. In two articles, no outcome evidence data were collected (21, 22).

Two of the narrative reviews (20, 23) were of high quality, as they had no methodological limitations. They also included primary studies relevant to the research question and the data collected were adequate and consistent with the objectives (Table 2). The included systematic reviews had very low quality (19, 24), mainly because the authors did not collect the reasons for exclusion, did not assess the risk of bias, and did not address publication bias (Table 3).

**Table 2:** Quality appraisal of the narrative reviews (GRADE-CERQual) (Spain, 2022).

	Methodological limitations	Coherence	Adequacy	Relevance	GRADE-CERQual
Ousager (18).	+	+/−	+/−	+	Moderate
Haidet (21).	+	+	+/−	+/−	Moderate
Carr (20).	+	+	+	+	High
Argawal (22).	+/−	+	+	+/−	Moderate
Perry (23).	+	+	+	+	High

**Table 3:** Quality appraisal of the systematic reviews (AMSTAR) (Spain, 2022).

	1	2	3	4	5	6	7	8	9	10	11	12	13	14	15	16	Confidence
Remein (19).	Y	N	N	P	Y	Y	N	N	N	N	Na	Na	Y	N	N	Y	Very low
Hoang (24).	Y	P	N	Y	Y	Y	N	P	N	N	Na	Na	N	N	Na	Y	Very low

Y, Yes; N, No; P, partially yes; Na, not applicable.

Table 4 shows those potentially relevant studies and the reasons for excluding them (9, 25–30).

**Table 4:** Excluded potentially relevant studies (Spain, 2022).

Studies	Reasons for exclusion
Barber et al. (25)	The objective was to generate a construct to evaluate social responsibility in medical education.
Brennan et al. (26)	Out of scope. It reported interventions to remedy failures in professionalism.
Lorenz et al. (27)	Not a review. Intervention to improve a more humane end-of-life care.
Raine (28)	It linked global health experiences to ethical issues.
Howick et al. (9)	It reported the inclusion of medical humanities in curricula.
Klein et al. (29)	Review to identify stressors in medical students and how to reduce them.
Moniz et al. (30)	Scoping review on the use of arts and humanities in medical education. Learning outcomes and their assessment were not covered.

## Discussion

The results of this review show that the most widely described medical humanities were philosophy, ethics, anthropology, and arts in several forms, and that there exists heterogeneity in teaching medical humanities regarding content and technical characteristics. Notwithstanding, and agreeing with Shapiro (1), several common characteristics were found, including using a methodology to investigate illness, teaching students to reflect on the surrounding reality, and encouraging collaboration between physicians and patients. Regarding the related expected outcome of promoting self-reflection, it was found that reflective writing was the most widely applied methodology. Furthermore, it meant a way of assessing the non-technical side of medical knowledge (31). In this sense, narrative medicine may enhance students’ understanding so that they can better recognize, understand, and interpret patients’ medical records and their health issues (32) by promoting more accurate scientific publications and presentations (33).

An assessment was also made of the impact of medical humanities on changes in behaviors and attitudes, relationship-building skills, development of empathy, personal achievement, and clinical skills, as well as on narrative competence and communication. Concerning the effect of humanities on students’ medical practice, the followed assessing methodology included the qualities of the humanities, students’ engagement, the acquisition of new knowledge, and its application to clinical practice (21). On this matter, observational skills were necessary for the diagnostic and therapeutic process, as it requires the integration of pieces of information into clinical judgment (3).

The learning outcomes described in this review can be classified into four clusters: (1) adequate communication (18–22); (2) development of skills to help in problem-solving (18, 24), capacity to reflect (19–21, 24), personal wellbeing and handling burnout (19, 20), coping with uncertainty and end-of-life care (21, 22), and developing critical and creative thinking (22, 24); (3) ethical compliance (18–20); and (4) teamwork (19). These results vary widely, which shows that there is currently no international consensus on what the learning outcomes of medical humanities should be.

**Figure 2:**
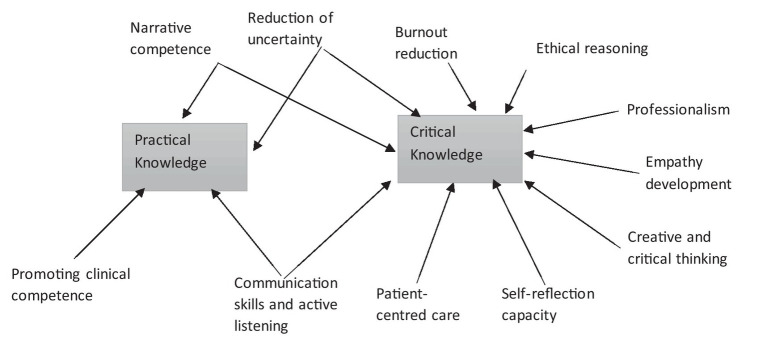
Correlation between medical humanities’ learning outcomes and types of knowledge (Spain, 2022).

Since medical humanities are integral parts of clinical practice and may be understood as “ways of knowing” (5), the different learning outcomes described in this review can be correlated with the types of knowledge proposed by Kumagai (6) (Figure 2). Most of these outcomes can be associated with critical/emancipatory knowledge (5), although some of them can be related to more than one type of knowledge. This is the case of narrative competence or communication skills, which could be linked to practical and critical knowledge; that is, complying with the standards of good practice (practical knowledge) and encouraging self-reflection (critical knowledge). This overlap is also observed in the “reduction of uncertainty” outcome, to which knowledge of clinical practice standards and the reflective process would contribute.

Acknowledging the learning outcomes of medical humanities from an epistemological perspective broadens their understanding and means a change in their assessment strategies. As Chiavaroli (5) pointed out, empathy, understood as a way of knowing, would imply understanding the circumstances surrounding both the patients and the disease to be able to apply the acquired knowledge in the clinical practice. Consequently, its assessment would focus on the quality of that understanding.

The amount of technical knowledge required for medical care may occasionally be small in comparison to other types of knowledge about human behavior which are part of medical humanities training (34). Therefore, considering humanities an enriching resource for bettering clinical practice may improve their acceptance by physicians (35). Notwithstanding this fact, the long-term impact of medical humanities curricula on medical students is yet underreported, even though Peters et al. (36) found that 10 years after the implementation of a medical humanities curriculum, professionals had better coping strategies for psychosocial problems.

In this review, training in humanities was given to undergraduate students. The implementation of humanities programs in health sciences degrees has taken place in recent years and many professionals have not had the opportunity to access undergraduate training in humanities. In the light of the important role they play in the understanding of the healthy and sick human being, it is also important to train professionals at postgraduate level through continuing education courses, with the aim of integrating technical and humanistic knowledge into their clinical practice (37).

Consequently, more empirical evidence of medical humanities learning outcomes is needed (18).

### Limitations

The present study has some limitations. Firstly, most of the studies were narrative and scoping reviews, although some were reported as systematic reviews (21, 22). Narrative reviews are more susceptible to bias, but their inclusion was considered relevant as they provided data from qualitative studies. Secondly, there may be a publication bias, affecting both the studies included in the reviews and the reviews themselves, since it is more common to publish studies that provide positive results.

An exhaustive bibliographic review was carried out by searching for information in several search engines such as Pubmed, ERIC, CINAHL, EMBASE, COCHRANE, and DARE, and on platforms such as WOS, which includes bibliographic databases and references to scientific publications in the field of medicine and the humanities, among others. However, some references may not be included in these databases and have therefore not been included in this review.

## Conclusion

This meta-review describes the learning outcomes of medical humanities curricula, including understanding of patients’ problems, improving communication with patients through narrative skills, and promoting empathy and ethical reasoning. In relation to the professionals themselves, medical humanities contribute to the development of skills to cope with uncertainty, promote creative thinking, and reduce burnout.

Humanities learning outcomes are part of the knowledge required for good clinical practice. As a result, the epistemological approach provides a valid argument for including the humanities in medical curricula. It would be advisable to improve the assessing methods of humanities curricula, standardizing key concepts to allow comparability, and enhance the evidence of results.

## Author contributions

VC-V, JG-S, CA-R, MR-D, and SÁ-M: conceptualization, data curation, formal analysis, investigation, methodology, resources, software, supervision, validation, visualization, writing – original draft, and writing – review and editing. VC-V and SÁ-M: project administration. All the authors have intellectually contributed to the work, met the conditions of authorship, and approved its final version.

## Conflict of interest

The authors declare that the research was conducted in the absence of any commercial or financial relationships that could be construed as a potential conflict of interest.

## Publisher’s note

All claims expressed in this article are solely those of the authors and do not necessarily represent those of their affiliated organizations, or those of the publisher, the editors and the reviewers. Any product that may be evaluated in this article, or claim that may be made by its manufacturer, is not guaranteed or endorsed by the publisher.

## References

[1] Shapiro J Coulehan J Wear D Montello M. Medical humanities and their discontents: definitions, critiques, and implications. Acad Med. (2009) 84:192–8. doi: 10.1097/ACM.0b013e3181938bca, PMID: 19174663

[2] Sánchez González MA. El humanismo y la enseñanza de las humanidades médicas. Educ Med. (2017) 18:212–8. doi: 10.1016/j.edumed.2017.03.001

[3] Shapiro J Rucker L Beck J. Training the clinical eye and mind: using the arts to develop medical students' observational and pattern recognition skills. Med Educ. (2006) 40:263–8. doi: 10.1111/j.1365-2929.2006.02389.x, PMID: 16483329

[4] Macnaughton J. The humanities in medical education: context, outcomes and structures. Med Humanit. (2000) 26:23–30. doi: 10.1136/mh.26.1.23 12484317

[5] Chiavaroli N. Knowing how we know: an epistemological rationale for the medical humanities. Med Educ. (2017) 51:13–21. doi: 10.1111/medu.13147, PMID: 27981654

[6] Kumagai AK. From competencies to human interests: ways of knowing and understanding in medical education. Acad Med. (2014) 89:978–3. doi: 10.1097/ACM.0000000000000234 24662200

[7] Habermas J. Knowledge and human interests. New York: John Wiley & Sons (2015).

[8] Berliner DC. Educational research: the hardest science of all. Educ Res. (2002) 31:18–20. doi: 10.3102/0013189X031008018

[9] Howick J Zhao L McKaig B Rosa A Campaner R Oke J. Do medical schools teach medical humanities? Review of curricula in the United States, Canada and the United Kingdom. J Eval Clin Pract. (2022) 28:86–92. doi: 10.1111/jep.13589, PMID: 34105226

[10] Doukas DJ McCullough LB Wear S. Project to rebalance and integrate medical education (PRIME) investigators. Perspective: medical education in medical ethics and humanities as the foundation for developing medical professionalism. Acad Med. (2012) 87:334–1. doi: 10.1097/ACM.0b013e318244728c 22373629

[11] Orefice C Pérez J Baños JE. The presence of humanities in the curricula of medical students in Italy and Spain. Educación médica. (2019) 20:79–86. doi: 10.1016/j.edumed.2017.10.008

[12] Columbia University Irving Medical Center. Division of narrative medicine. Columbia, NY, USA: Department of Medical Humanities and Ethics (2020).

[13] Bologna Working Group on Qualifications Frameworks. Qualifications: A framework for qualifications of the European higher education area. Copenhagen: Ministry of Science, Technologie and Innovation (2005).

[14] Agencia Nacional de la Evaluación de la Calidad y Acreditación (ANECA). Guía de apoyo para la redacción, puesta en práctica y evaluación de los resultados del aprendizaje. Madrid: ANECA (2017). 68 p.

[15] Moher D Liberati A Tetzlaff J Altman DG PRISMA Group. Preferred reporting items for systematic reviews and meta-analyses: the PRISMA statement. PLoS Med. (2009) 6:e1000097. 19621072 10.1371/journal.pmed.1000097 PMC2707599

[16] Lewin S Booth A Glenton C Munthe-Kaas H Rashidian A Wainwright M. Applying GRADE-CERQual to qualitative evidence synthesis findings: introduction to the series. Implement Sci. (2018) 13:2. doi: 10.1186/s13012-017-0688-3, PMID: 29384079 PMC5791040

[17] Shea BJ Reeves BC Wells G Thuku M Hamel C Moran J. AMSTAR 2: a critical appraisal tool for systematic reviews that include randomised or non-randomised studies of healthcare interventions, or both. BMJ. (2017) 21:j4008. doi: 10.1136/bmj.j4008 PMC5833365 28935701

[18] Ousager J Johannessen H. Humanities in undergraduate medical education: a literature review. Acad Med. (2010) 85:988–8. doi: 10.1097/ACM.0b013e3181dd226b 20505399

[19] Remein CD Childs E Pasco JC Trinquart L Flynn DB. Content and outcomes of narrative medicine programmes: a systematic review of the literature through 2019. BMJ Open. (2019) 10:e031568. doi: 10.1136/bmjopen-2019-031568, PMID: 31988222 PMC7045204

[20] Carr SE Noya F Phillips B Harris A Scott K Hooker C. Health humanities curriculum and evaluation in health professions education: a scoping review. BMC Med Educ. (2021) 21:568. doi: 10.1186/s12909-021-03002-1, PMID: 34753482 PMC8579562

[21] Haidet P Jarecke J Adams NE Stuckey HL Green MJ Shapiro D. A guiding framework to maximise the power of the arts in medical education: a systematic review and metasynthesis. Med Educ. (2016) 50:320–1. doi: 10.1111/medu.12925, PMID: 26896017

[22] Agarwal A Wong S Sarfaty S Devaiah A Hirsch AE. Elective courses for medical students during the preclinical curriculum: a systematic review and evaluation. Med Educ Online. (2015) 20:26615. doi: 10.3402/meo.v20.26615 25968131 PMC4429260

[23] Perry M Maffulli N Willson S Morrissey D. The effectiveness of arts-based interventions in medical education: a literature review. Med Educ. (2011) 45:141–8. doi: 10.1111/j.1365-2923.2010.03848.x, PMID: 21208260

[24] Hoang BL Monrouxe LV Chen KS Chang SC Chiavaroli N Mauludina YS. Medical humanities education and its influence on Students' outcomes in Taiwan: a systematic review. Front Med (Lausanne). (2022) 9:857488. doi: 10.3389/fmed.2022.857488, PMID: 35652071 PMC9150274

[25] Barber C van der Vleuten C Leppink J Chahine S. Social accountability frameworks and their implications for medical education and program evaluation: a narrative review. Acad Med. (2020) 95, 12:1945–54. doi: 10.1097/ACM.0000000000003731, PMID: 32910000

[26] Brennan N Price T Archer J Brett J. Remediating professionalism lapses in medical students and doctors: a systematic review. Med Educ. (2020) 54:196–4. doi: 10.1111/medu.14016, PMID: 31872509

[27] Lorenz KA Steckart MJ Rosenfeld KE. End-of-life education using the dramatic arts: the wit educational initiative. Acad Med. (2004) 79:481–6. doi: 10.1097/00001888-200405000-00020, PMID: 15107289

[28] Raine SP. Ethical issues in education: medical trainees and the global health experience. Best Pract Res Clin Obstet Gynaecol. (2017) 43:115–4. doi: 10.1016/j.bpobgyn.2017.03.004 28410992

[29] Klein HJ McCarthy SM Sarah M. Student wellness trends and interventions in medical education: a narrative review. Human. Soc. Sci. Commun. (2022) 9

[30] Moniz T Golafshani M Gaspar CM Adams NE Haidet P Sukhera J. How are the arts and humanities used in medical education? Results of a scoping review. Acad Med. (2021) 96:1213–22. doi: 10.1097/ACM.0000000000004118, PMID: 33830951

[31] Meier DE Back AL Morrison RS. The inner life of physicians and care of the seriously ill. JAMA. (2001) 286:3007–14. doi: 10.1001/jama.286.23.3007, PMID: 11743845

[32] Caronte R Medicina narrativa. Un modelo de empatía, reflexión, profesión y confianza. JAMA. (2001) 286:1897–02.11597295

[33] Steinert Y McLeod PJ Liben S. Writing for publication in medical education: the benefits of a faculty development workshop and peer writing group. Med Teach. (2008) 30:e280–5. doi: 10.1080/01421590802337120, PMID: 18946816

[34] Edmondson R Pearce J. The practice of health care: wisdom as a model. Med Health Care Philos. (2007, 2007) 10:233–44. 17120113 10.1007/s11019-006-9033-3

[35] Knight LV. A silly expression: consultants' implicit and explicit understanding of medical humanities. Qual Anal Med Human. (2006) 32:119–4. doi: 10.1136/jmh.2006.000238, PMID: 23673809

[36] Peters AS Greenberger-Rosovsky R Crowder C Block SD Moore GT. Long-term outcomes of the new pathway program at Harvard Medical School: a randomized controlled trial. Acad Med. (2000) 75:470–9. doi: 10.1097/00001888-200005000-00018, PMID: 10824772

[37] O'Neill D Kelly B O'Keeffe S Moss H. Mainstreaming medical humanities in continuing professional development and postgraduate training. Clin Med (Lond). (2020) 20:208–1. doi: 10.7861/clinmed.2019-0332, PMID: 32188660 PMC7081798

